# Comparative analysis of Denosumab and Zoledronic acid in advanced breast cancer patients receiving CDK4/6 inhibitors

**DOI:** 10.1016/j.breast.2025.104502

**Published:** 2025-05-15

**Authors:** Roberta Scafetta, Marco Donato, Carla Gullotta, Alessandra Guarino, Cristina Fiore, Luisana Sisca, Elena Speziale, Raffaella Troiano, Simone Foderaro, Valentina Ricozzi, Michele Iuliani, Sonia Simonetti, Silvia Cavaliere, Alessio Cortellini, Andrea Botticelli, Simone Scagnoli, Simona Pisegna, Carmen Criscitiello, Rebecca Pedersini, Caterina Sposetti, Elisa Tiberi, Giuliana D'Auria, Matteo Vergati, Marco Mazzotta, Roberta Caputo, Annarita Verrazzo, Maria Grazia Rossino, Federica Domati, Claudia Piombino, Francesca Sofia Di Lisa, Lorena Filomeno, Teresa Arcuri, Federica Puce, Federica Riva, Michela Palleschi, Marianna Sirico, Marta Piras, Luigia Stefania Stucci, Delia De Lisi, Paolo Orsaria, Edy Ippolito, Sara Ramella, Luca Visani, Niccolò Bertini, Ilaria Bonaparte, Stefania Gori, Luigi Rossi, Icro Meattini, Barbara Tagliaferri, Orazio Caffo, Ilaria Portarena, Azzurra Irelli, Elisabetta Cretella, Camillo Porta, Giampaolo Bianchini, Agnese Fabbri, Ugo De Giorgi, Patrizia Vici, Angela Toss, Ornella Garrone, Michelino De Laurentiis, Federica Villa, Rossana Berardi, Mauro Minelli, Claudio Vernieri, Giuseppe Curigliano, Bruno Vincenzi, Giuseppe Tonini, Daniele Santini, Francesco Pantano

**Affiliations:** a Medical Oncology, Fondazione Policlinico Universitario Campus Bio-Medico, Via Alvaro del Portillo 200, 00128, Roma, Italy; b Medical Oncology Unit, Central Hospital of Belcolle, Department of Oncology and Hematology, Strada Sammartinese snc, 01100, Viterbo, Italy; c Department of Medicine and Surgery, Università Campus Bio-Medico di Roma, Via Alvaro del Portillo 21, 00128, Roma, Italy; d Dip. Scienze e Biotecnologie Medico-chirurgiche, Policlinico Umberto I, “Sapienza” University of Rome, Rome, Italy; e Division of Early Drug Development for Innovative Therapy, IEO, European Institute of Oncology, IRCCS, Milan, Italy; f Department of Oncology and Hemato-Oncology, University of Milano, Milano, Italy; g Medical Oncology Department, ASST Spedali Civili of Brescia, Brescia, Italy; h Department of Medical Oncology, Fondazione IRCCS Istituto Nazionale dei Tumori, Milan, Italy; i Department of Medical Oncology, Università Politecnica Delle Marche, AOU delle Marche, Ancona, Italy; j Department of Medical Oncology, Medical Oncology Unit, Sandro Pertini Hospital, Rome, Italy; k Department of Breast and Thoracic Oncology, Division of Breast Medical Oncology, Istituto di Ricovero e Cura a Carattere Scientifico (IRCCS) Pascale, Naples, Italy; l Medical Oncology Fondazione IRCCS Ca’ Granda Ospedale Maggiore Policlinico, 20122, Milan, Italy; m Department of Oncology and Hematology, Azienda Ospedaliero-Universitaria di Modena, Modena, Italy; n UOSD Sperimentazioni di fase IV - IRCCS Istituto Nazionale Tumori Regina Elena, Via Elio Chianesi 53, 00144, Roma, Italy; o Medical Oncology Unit, ICS Maugeri IRCCS, Pavia, Italy; p Department of Clinical and Molecular Medicine, "La Sapienza" University of Rome, Policlinico Umberto I, Viale Regina Elena 324, 00161, Rome, Italy; q Department of Medical Oncology, IRCCS Istituto Romagnolo per lo Studio dei Tumori (IRST) “Dino Amadori”, Meldola, Italy; r Department of Medical Oncology, IRCCS San Raffaele Scientific Institute, Milan, Italy; s Division of Medical Oncology, A.O.U. Consorziale Policlinico Di Bari, Bari, Italy; t Medical Oncology Unit, Santa Chiara Hospital, APSS Trento, Italy; u Department of Breast Surgery, Fondazione Policlinico Universitario Campus Bio-Medico, Rome, Italy; v Department of Radiation Oncology (Medicine and Surgery), Università Campus Bio-Medico di Roma, Rome, Italy; w Radiation Oncology, Fondazione Policlinico Universitario Campus Bio-Medico, Rome, Italy; x Radiation Oncology Unit & Breast Unit, Azienda Ospedaliero-Universitaria Careggi, University of Florence, Florence, Italy; y Medical Oncology, IRCCS Ospedale Sacro Cuore Don Calabria, Negrar di Valpolicella (VR), Italy; z UOC of Oncology - ASL Latina - Distretto 1, University of Rome “Sapienza”, Formia, Italy; aa Department of Experimental and Clinical Biomedical Sciences, University of Florence, Florence, Italy; ab Department of Oncology, Policlinico Tor Vergata University, Rome, Italy; ac Medical Oncology Unit, Department of Oncology, “Giuseppe Mazzini” Hospital, AUSL 04 Teramo, 64100, Teramo, Italy; ad Medical Oncology Unit, Ospedale di Bolzano, Bolzano, Italy; ae Università Vita-Salute San Raffaele, Milan, Italy; af Medical Oncology, Oncology Department ASST Lecco, 23900, Lecco, Italy; ag UOC Oncologia, Azienda Ospedaliera San Giovanni Addolorata, Roma, Italy

**Keywords:** Bone metastasis, Breast cancer, Denosumab, Zoledronic acid, CDK4/6 inhibitors

## Abstract

A comparative analysis of Denosumab (DMAB) and Zoledronic Acid (ZA) was conducted in a real-world cohort of 864 patients with hormone receptor-positive, human epidermal growth factor receptor 2-negative advanced breast cancer with bone metastases, who were undergoing CDK4/6 inhibitors plus endocrine therapy. We evaluated the time to first skeletal-related events (SREs), progression-free survival (PFS), and overall survival (OS). To adjust for confounding variables, we utilized propensity score matching (PSM) and inverse probability of treatment weighting (IPTW) methodologies. In the unadjusted cohort, ZA was associated with a longer time to first SRE compared to DMAB (HR = 0.77, 95 % CI: 0.61–0.98, p = 0.031). Similar results were obtained in both the PSM (HR = 0.69, 95 % CI: 0.52–0.92, p = 0.011) and IPTW cohorts (HR = 0.74, 95 % CI: 0.63–0.87, p < 0.001), with ZA-treated patients showing an extended time to first SRE compared to those treated with DMAB. No differences in PFS and OS were observed between the two cohorts.

## Highlights

• Real world study of 864 advanced breast cancer patients receiving CDK4/6 inhibitors.
• Zoledronic acid shows superiority over denosumab in delaying time to first SRE.
• No difference in PFS or OS observed between the treatment groups.

## 1 Introduction

Breast cancer (BC) is a major health concern worldwide, being the most common cancer among women and a leading cause of cancer-related deaths [1]. One of the most challenging aspects of managing advanced BC (aBC) is the high incidence of bone metastases [2]. These metastases are not only common but also significantly debilitating, leading to skeletal-related events (SREs) such as pathologic fractures, spinal cord compression, radiation or surgery to bone, and hypercalcemia of malignancy. These complications drastically reduce the quality of life for patients and increase healthcare costs [3].

Two primary therapies used to manage and prevent SREs in aBC patients with bone metastases are Zoledronic acid (ZA) and Denosumab (DMAB) [4]. Both drugs work by inhibiting osteoclast-mediated bone resorption but through different mechanisms [5]. ZA is a third-generation bisphosphonate that integrates into the bone matrix and has a long half-life. It is administered intravenously and works by inhibiting osteoclast activity, thereby reducing the risk of fractures, spinal cord compression, and the need for bone radiation or surgery [6,7]. ZA also exhibits antitumor properties by inducing apoptosis in cancer cells, inhibiting angiogenesis, and preventing tumor cell adhesion to the bone matrix [8]. DMAB is a monoclonal antibody that targets RANKL, a key molecule involved in osteoclast formation and function [9]. DMAB is administered subcutaneously and acts more rapidly than ZA. It can be cleared from the circulation without being retained in the bone, which has implications for patient management, particularly in terms of renal function and the risk of osteonecrosis of the jaw (ONJ) [9]. While ZA is associated with renal toxicity and requires monitoring of kidney function, DMAB has a higher risk of hypocalcemia and ONJ, necessitating careful patient selection and monitoring [5].

The treatment landscape for HR+/HER2-aBC has been revolutionized by the introduction of cyclin-dependent kinases (CDK) 4/6 inhibitors (CDK4/6i) in combination with standard endocrine therapy (ET). These therapies have significantly improved progression-free survival (PFS) and overall survival (OS) in patients, including those with bone metastases [[10], [11], [12], [13], [14], [15], [16], [17]]. CDK4/6 inhibitors work by slowing down tumor progression and enhancing tumor responses in the bone. However, they also prolong patient exposure to metastatic disease processes, which raises the question of how ZA and DMAB might differently affect the risk of SREs and disease progression in this context.

Despite the known benefits of ZA and DMAB, there is a lack of comprehensive data comparing their effects specifically in aBC patients treated with CDK4/6i plus ET. Prospective randomized registration trials have demonstrated that DMAB is more effective than ZA in delaying or preventing SREs in patients with bone metastatic BC who are treated with chemotherapy or ET [[18], [19], [20]]. Specifically, the Phase III study by Stopeck et al. demonstrated a statistically significant improvement in SRE control with DMAB over ZA [18]. However, the clinical significance of this difference can be questioned, as the absolute reduction in SREs is modest. Additionally, it is important to note that studies, including Stopeck et al., have not shown that bone-modifying agents (BMA) impact OS.

Since CDK4/6i have shown significant efficacy in improving survival endpoints, their combination with BMAs could potentially enhance these benefits by better controlling bone metastases and reducing SREs, thereby improving overall patient outcomes. This study aims to fill this knowledge gap by retrospectively analyzing the comparative effects of ZA and DMAB on SREs and disease progression in a cohort of BC patients with bone metastases receiving CDK4/6i plus ET. By understanding the differential impacts of these bone-modifying agents in the context of CDK4/6 inhibition, the research seeks to provide insights that could lead to better clinical decision-making and improved outcomes for patients with advanced BC.

## 2 Results

### 2.1 Patient characteristics

We enrolled 864 HR+/HER2-aBC patients with bone metastases, and treated with ET plus CDK4/6i, as first- or second-line of therapy. 618 patients (71.53 %) received DMAB and 246 (28.47 %) were treated with ZA. When compared to patients treated with DMAB, patients receiving ZA were more likely to have poorer ECOG PS (1 vs. 0), to receive CDK4/6i as a second line of therapy, to have endocrine-resistant disease and to receive fulvestrant as an ET (Table 1). On the other hand, patients receiving DMAB were more likely to have bone-only disease (Table 1). Finally, there were no significant imbalances, in terms of the specific CDK4/6i received, between patients treated with ZA or DMAB. Following propensity score matching (PSM), these baseline differences were mitigated, finally resulting in successfully matched cohorts (Table 2). Similarly, after inverse probability of treatment weighting (IPTW) adjustment, the distributions of clinic-pathological characteristics were balanced between the DMAB and ZA arms (Table 3).

**Table 1:** Clinic-pathological features of the “unadjusted” cohort.

Characteristics		Denosumab	Zoledronic Acid	p *value*	SMD
**n**		618	246		
**Premenopausal State**	No	460 (74.4 %)	194 (78.9 %)	0.2	0.105
	Yes	158 (25.6 %)	52 (21.1 %)		
**Age (mean** ± **SD)**		60.1 ± 12.7	61.0 ± 12.0	0.328	0.073
**PS**	**0**	**556 (90.0 %)**	**201 (81.7 %)**	**0.001**	**0.239**
	**1**	**62 (10.0 %)**	**45 (18.3 %)**		
**Histology**	Ductal	443 (71.7 %)	165 (67.1 %)	0.405	0.101
	Lobular	133 (21.5 %)	61 (24.8 %)		
	Other	42 (6.8 %)	20 (8.1 %)		
**Ki67 (mean** ± **SD)**		23.5 ± 14.8	21.4 ± 15.0	0.079	0.139
**Grading**	G1-G2	294 (64.5 %)	131 (66.8 %)	0.623	0.05
	G3	162 (35.5 %)	65 (33.2 %)		
**Estrogen Receptor (mean** ± **SD)**		86.7 ± 15.2	86.8 ± 14.7	0.926	0.007
**Progesterone Receptor (mean ± SD)**		46.4 ± 36.6	48.0 ± 36.2	0.575	0.046
**Metastatic at Diagnosis**	No	353 (57.2 %)	134 (54.5 %)	0.511	0.055
	Yes	264 (42.8 %)	112 (45.5 %)		
**Adjuvant Chemotherapy**	No	364 (60.0 %)	153 (63.0 %)	0.465	0.062
	Yes	243 (40.0 %)	90 (37.0 %)		
**Adjuvant Endocrine Therapy**	No	227 (39.4 %)	93 (40.6 %)	0.815	0.025
	Yes	349 (60.6 %)	136 (59.4 %)		
**Months of Adjuvant Endocrine Therapy (mean** ± **SD)**		46.6 ± 30.1	41.6 ± 30.8	0.092	0.166
**Bone-only disease**	**No**	**305 (49.4 %)**	**84 (34.1 %)**	**<0.001**	**0.312**
	**Yes**	**313 (50.6 %)**	**162 (65.9 %)**		
**CDK4/6 Inhibitor**	Abemaciclib	86 (13.9 %)	37 (15.0 %)	0.096	0.166
	Palbociclib	338 (54.7 %)	150 (61.0 %)		
	Ribociclib	194 (31.4 %)	59 (24.0 %)		
**Line of Treatment**	**First**	**507 (84.4 %)**	**171 (70.1 %)**	**<0.001**	**0.345**
	**Second**	**94 (15.6 %)**	**73 (29.9 %)**		
**Setting**	**Endocrine Resistant**	**245 (39.6 %)**	**121 (49.2 %)**	**0.013**	**0.193**
	**Endocrine Sensitive**	**373 (60.4 %)**	**125 (50.8 %)**		
**Endocrine Therapy**	**Aromatase Inhibitor**	**397 (64.2 %)**	**138 (56.1 %)**	**0.032**	**0.167**
	**Fulvestrant**	**221 (35.8 %)**	**108 (43.9 %)**

**Table 2:** Clinic-pathological features of the “PSM” cohort.

Characteristics		Denosumab	Zoledronic Acid	p *value*	SMD
**n**		226	226		
**Premenopausal State**	No	179 (79.2 %)	180 (79.6 %)	1	0.011
	Yes	47 (20.8 %)	46 (20.4 %)		
**Age (mean ± SD)**		62.4 ± 12.1	61.5 ± 11.7	0.41	0.078
**PS**	0	189 (83.6 %)	187 (82.7 %)	0.9	0.024
	1	37 (16.4 %)	39 (17.3 %)		
**Histology**	Ductal	158 (69.9 %)	150 (66.4 %)	0.71	0.078
	Lobular	51 (22.6 %)	58 (25.7 %)		
	Other	17 (7.5 %)	18 (8.0 %)		
**Ki67 (mean ± SD)**		21.5 ± 14.1	21.7 ± 14.9	0.895	0.013
**Grading**	G1-G2	99 (62.3 %)	122 (66.7 %)	0.462	0.092
	G3	60 (37.7 %)	61 (33.3 %)		
**Estrogen Receptor (mean ± SD)**		85.2 ± 16.1	86.7 ± 14.9	0.313	0.101
**Progesterone Receptor (mean ± SD)**		45.7 ± 38.2	48.6 ± 36.0	0.438	0.078
**Metastatic at Diagnosis**	No	135 (59.7 %)	120 (53.1 %)	0.184	0.134
	Yes	91 (40.3 %)	106 (46.9 %)		
**Adjuvant Chemotherapy**	No	148 (65.5 %)	144 (63.7 %)	0.768	0.037
	Yes	78 (34.5 %)	82 (36.3 %)		
**Adjuvant Endocrine Therapy**	No	87 (38.5 %)	91 (40.3 %)	0.773	0.036
	Yes	139 (61.5 %)	135 (59.7 %)		
**Months of Adjuvant Endocrine Therapy (mean ± SD)**		47.6 ± 26.2	41.8 ± 31.2	0.099	0.202
**BoneOnly**	No	92 (40.7 %)	76 (33.6 %)	0.144	0.147
	Yes	134 (59.3 %)	150 (66.4 %)		
**CDK4/6 Inhibitor**	Abemaciclib	29 (12.8 %)	31 (13.7 %)	0.791	0.064
	Palbociclib	145 (64.2 %)	138 (61.1 %)		
	Ribociclib	52 (23.0 %)	57 (25.2 %)		
**Line of Treatment**	First	154 (68.1 %)	159 (70.4 %)	0.683	0.048
	Second	72 (31.9 %)	67 (29.6 %)		
**Setting**	Endocrine Resistant	118 (52.2 %)	111 (49.1 %)	0.572	0.062
	Endocrine Sensitive	108 (47.8 %)	115 (50.9 %)		
**Endocrine Therapy**	Aromatase Inhibitor	134 (59.3 %)	128 (56.6 %)	0.634	0.054
	Fulvestrant	92 (40.7 %)	98 (43.4 %)

**Table 3:** Clinic-pathological features of the “IPTW” cohort.

Characteristics		Denosumab	Zoledronic Acid	p *value*	SMD
**n**		617	759		
**Premenopausal State**	No	476 (77.1 %)	606 (79.8 %)	0.252	0.066
	Yes	141 (22.9 %)	153 (20.2 %)		
**Age (mean ± SD)**		61.4 ± 12.4	61.5 ± 11.6	0.905	0.007
**PS**	0	529 (85.7 %)	665 (87.6 %)	0.346	0.055
	1	88 (14.3 %)	94 (12.4 %)		
**Histology**	Ductal	432 (70.0 %)	520 (68.5 %)	0.799	0.036
	Lobular	137 (22.2 %)	180 (23.7 %)		
	Other	48 (7.8 %)	59 (7.8 %)		
**Ki67 (mean ± SD)**		22.7 ± 14.4	21.8 ± 14.4	0.271	0.062
**Grading**	G1-G2	292 (64.6 %)	406 (65.2 %)	0.899	0.012
	G3	160 (35.4 %)	217 (34.8 %)		
**Estrogen Receptor (mean ± SD)**		86.1 ± 15.7	86.2 ± 15.4	0.899	0.007
**Progesterone Receptor (mean ± SD)**		45.8 ± 37.2	46.3 ± 36.1	0.817	0.014
**Metastatic at Diagnosis**	No	351 (56.9 %)	399 (52.6 %)	0.122	0.087
	Yes	266 (43.1 %)	360 (47.4 %)		
**Adjuvant Chemotherapy**	No	381 (61.8 %)	480 (63.2 %)	0.608	0.031
	Yes	236 (38.2 %)	279 (36.8 %)		
**Adjuvant Endocrine Therapy**	No	245 (39.7 %)	298 (39.3 %)	0.91	0.009
	Yes	372 (60.3 %)	461 (60.7 %)		
**Months of Adjuvant Endocrine Therapy (mean ± SD)**		48.2 ± 29.1	44.4 ± 31.2	0.075	0.125
**BoneOnly**	No	285 (46.2 %)	364 (48.0 %)	0.549	0.035
	Yes	332 (53.8 %)	395 (52.0 %)		
**CDK4/6 Inhibitor**	Abemaciclib	85 (13.8 %)	98 (12.9 %)	0.837	0.032
	Palbociclib	352 (57.0 %)	444 (58.5 %)		
	Ribociclib	180 (29.2 %)	217 (28.6 %)		
**Line of Treatment**	First	485 (78.6 %)	609 (80.2 %)	0.498	0.04
	Second	132 (21.4 %)	150 (19.8 %)		
**Setting**	Endocrine Resistant	270 (43.8 %)	328 (43.2 %)	0.882	0.011
	Endocrine Sensitive	347 (56.2 %)	431 (56.8 %)		
**Endocrine Therapy**	Aromatase Inhibitor	389 (63.0 %)	462 (60.9 %)	0.441	0.045
	Fulvestrant	228 (37.0 %)	297 (39.1 %)		

### 2.2 Clinical outcomes of patients receiving ZA or DMAB

Median patient follow-up was 54.6 months (95 % CI: 50.0–64.0); median of the CDK4/6i and BMA treatment was 39.0 months (95 % CI: 37.0–42.0). Patient outcomes were evaluated across these three cohorts - referred to as “unadjusted,” “PSM,” and “IPTW” - in terms of time to first SRE, PFS and OS.

**Fig. 1:**
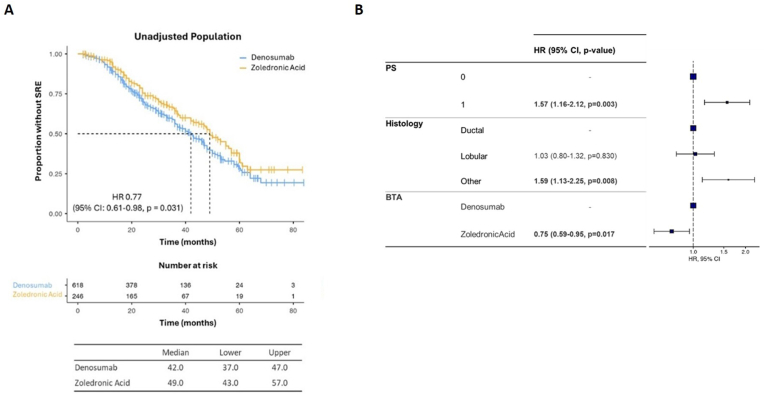
(A) Kaplan-Meier estimates of time to first SRE in “unadjusted” cohort. (B) Cox regression multivariate analysis.

In the “unadjusted” cohort, ZA is associated with significantly longer time to the occurrence of the first SRE (median 49.0 months; 95 % CI: 43.0–57.0) when compared to DMAB (median 42 months; 95 % CI: 37.0–47.0) (HR = 0.77, 95 % CI: 0.61–0.98, p = 0.031) (Fig. 1A).

In Cox Regression multivariate models we adjusted the association between ZA or DMAB with the risk of undergoing SREs for ECOG PS and tumor histology, the only variables significantly associated with time to first SRE at univariate analysis (**Supplementary Table**). These analyses confirmed a lower risk for time to first SRE in patients receiving ZA compared to those treated with DMAB (HR = 0.76, 95 % CI: 0.60–0.97, p = 0.024) (Fig. 1B).

**Fig. 2:**
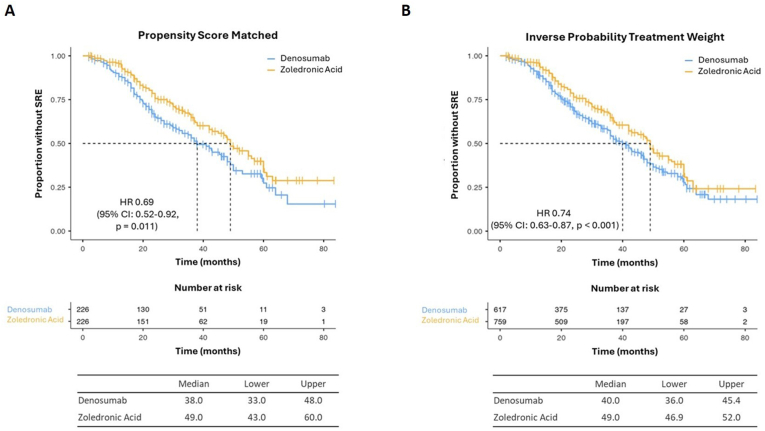
Kaplan-Meier estimates of time to first SRE in “PSM” (A) and “IPTW” (B) cohorts.

In the “PSM” cohort, patients treated with ZA exhibited an increased time to first SRE (median 49.0 months; 95 % CI: 43.0–60.0) compared to the DMAB group (median 38 months; 95 % CI: 33.0–48.0) (HR = 0.69, 95 % CI: 0.52–0.92, p = 0.011) (Fig. 2A). The “IPTW” cohort confirmed these findings, showing that patients treated with ZA experienced a longer time to first SRE (median 49.0 months; 95 % CI: 46.9–52.0) when compared to patients treated with DMAB (median 40.0 months; 95 % CI: 36.9–45.4) (HR = 0.74, 95 % CI: 0.63–0.87, p < 0.001) (Fig. 2B).

**Fig. 3:**
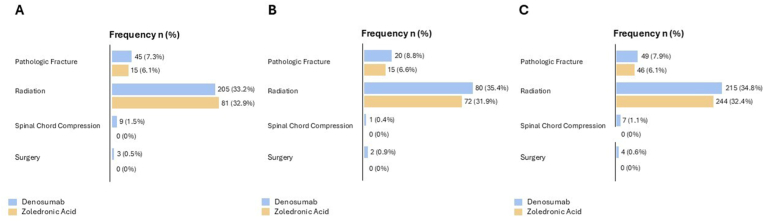
Frequency (%) of SRE type in “unadjusted” (A), “PSM” (B) and “IPTW” (C) cohorts.

No difference in SRE type was detected across the three cohort (Fig. 3).

**Fig. 4:**
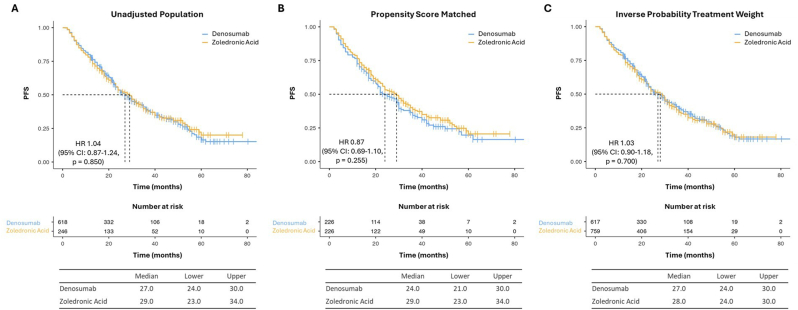
Kaplan-Meier estimates of PFS in “unadjusted” (A), “PSM” (B) and “IPTW” (C) cohorts.

**Fig. 5:**
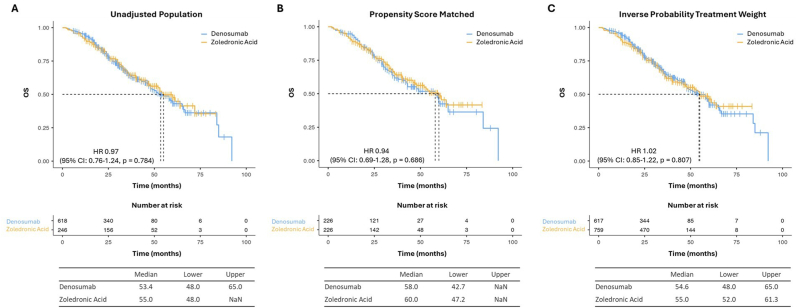
Kaplan-Meier estimates of OS in “unadjusted” (A), “PSM” (B) and “IPTW” (C) cohorts.

We found no significant PFS or OS differences between patients treated with ZA or DMAB (Fig. 4, Fig. 5).

## 3 Discussion

The management of bone metastases in BC patients is a critical aspect of oncology care, and it primarily aims at mitigating the potentially debilitating consequences of SREs. We conducted the most extensive Italian retrospective study to date, providing valuable insights into the comparative effectiveness of ZA and DMAB in combination with ET plus CDK4/6i, which represents the standard-of care therapy for patients with HR+/HER2-aBC. By assessing the role of BMA in a real-world population, we demonstrated the superiority of ZA over DMAB in delaying time to first SRE. Interestingly, PFS and OS were comparable between in the ZA and DMAB cohorts, which suggests that delaying the onset of SREs does not translate into a survival benefit in this clinical context. The use of PSM and IPTW methodologies allowed for a robust comparison by balancing clinic-pathological characteristics between the treatment groups, thereby enhancing the validity of our results.

This observation runs counter to all pivotal randomised phase III trials conducted in patients with aBC, in which DMAB consistently outperformed ZA [[18], [19], [20]]. Moreover, DMAB reduced the risk of both first and subsequent SREs in other tumour types. In the castration-resistant setting with established bone metastases, the pivotal randomised trial led by Fizazi, DMAB prolonged the median time to first SRE from 17.1 to 20.7 months, representing a relative risk reduction of approximately 18 % [21]. DMAB superiority has also been reported in subsequent meta-analyses, which show a pooled hazard ratio of 0.86 for DMAB versus ZA in delaying the first SRE [22]. Additional evidence confirmed the non-inferiority of DMAB in advanced cancers (excluding breast and prostate cancer) and multiple myeloma [23].

Unlike these trials, our study included patients receiving CDK4/6i plus ET that could influence the efficacy of BMAs in delaying the time to first SRE. Previous evidences showed that CDK4/6i induce cell cycle arrest, and they can affect the differentiation of non-tumor cells, such as osteoclasts [[24], [25], [26], [27], [28]]. Preclinical data from our group showed that CDK4/6i inhibit osteoclast differentiation and the expression of bone resorption markers, supporting the notion that cell cycle progression is crucial for osteoclast precursor differentiation [28].

The observed superiority of ZA over DMAB in preventing SREs may depend on the different mechanism through which these drugs act on bone metastases. ZA, a nitrogen-containing bisphosphonate, concentrates within mineralised bone and induces osteoclast apoptosis through mevalonate-pathway blockade. Beyond its anti-resorptive activity, ZA accumulates isopentenyl-pyrophosphate in tumour cells, thereby activating γδ T lymphocytes and other innate-immune effectors, and modulates angiogenic cytokines in the bone microenvironment [29]. CDK4/6 inhibition may therefore create a milieu in which ZA's immunomodulatory and anti-tumour properties add to, rather than merely duplicate, osteoclast suppression—whereas denosumab, a pure RANK-ligand inhibitor without direct tumour or immune effects, confers no such extra advantage. The selective benefit of adjuvant bisphosphonates, but not adjuvant DMAB, in improving the outcome in early breast cancer [30] supports the notion that ZA's pleiotropic mechanism can translate into clinical benefit when osteoclast activity is already partially dampened by other therapies.

However, the precise mechanisms responsible for the apparent synergy between CDK4/6i and ZA remain unclear, and dedicated mechanistic studies are still required.

The results of your study align with the previous studies that reported no significant differences in PFS and OS between the DMAB and ZA groups. This consistency suggests that while there may be differences in time to first SRE, survival outcomes remain similar.

While our findings are significant, it is important to recognize few limitations. The retrospective design may introduce biases inherent to observational studies. In addition, variability in the duration of exposure to BMAs could affect the comparative effectiveness of ZA and DMAB; the exact time on treatment for each agent was not captured, precluding exposure–response analyses. Nevertheless, per inclusion criteria, BMA therapy was continued in every patient until CDK4/6i discontinuation, ensuring a consistent treatment window across the cohort. Moreover, we did not collect data concerning adverse events associated with each treatment, including ONJ, because it was beyond the prespecified objectives.

In conclusion, our study provides valuable evidence supporting the superiority of ZA over DMAB HR+.HER2-aBC patients treated with ET plus CDK4/6i. This suggests that ZA may be more effective in delaying bone complications in this specific patient population. Additionally, the inclusion of CDK4/6i in the treatment regimen appears to influence the efficacy of BMAs. This highlights the potential synergistic effects of combining ZA with CDK4/6i, which could lead to better management of bone health in patients with bone metastases. However, future research should delve deeper into the mechanisms by which ZA and DMAB interact with CDK4/6 inhibitors and their long-term effects on patient survival and quality of life.

## 4 Methods

### 4.1 Study design

We conducted a retrospective analysis of data from 864 patients with HR+/HER2-aBC who were treated with CDK4/6 inhibitors (CDK4/6i) plus endocrine therapy (ET), either as first or second-line treatment, in combination with ZA or DMAB. Eligible patients had bone metastases at the diagnosis of advanced disease, an Eastern Cooperative Oncology Group (ECOG) Performance Status (PS) of 0 or 1, and a minimum follow-up duration of 12 months. We included both primary and secondary endocrine-resistant patients, defined as follows: Primary endocrine resistance is characterized by relapse within 2 years of adjuvant endocrine treatment or disease progression during the first 6 months of first-line endocrine therapy for aBC. Secondary resistance in early BC is defined as a relapse occurring after at least 2 years of endocrine therapy and during or within the first year of completing adjuvant endocrine therapy. In aBC, secondary resistance is defined as disease progression after more than 6 months of endocrine therapy.

Data collection spanned from January 2019 to December 2023. Ethical approval was granted by ethics committee of the coordination center, Fondazione Policlinico Universitario Campus Bio-Medico (PAR 30.22 OSS). Study was conducted in accordance with the Declaration of Helsinki. Informed consent was obtained only from patients who were alive at the time of the analysis. The collection of data from deceased patients was authorized by the Data Protection Officer of the respective centers.

The study population was identified through a review of medical records at the participating centers. Eligible patients had HR-positive/HER2-negative advanced breast cancer with bone metastases and received endocrine therapy plus a CDK4/6i together with either monthly ZA or monthly DMAB. Patients qualified only if the BMA was started concomitantly with the CDK4/6i or, at the latest, within three months of CDK4/6i initiation. Participants who switched from one BMA to another, delayed BMA administration for more than three months at any time during follow-up, began their BMA more than three months after starting the CDK4/6i, or discontinued the BMA before stopping CDK4/6i therapy were excluded.

SREs were identified through a comprehensive review of patient medical records, imaging studies, and clinical reports. These included both symptomatic events and incidentally found radiographic events. Symptomatic SREs were defined as those events that presented with clinical symptoms such as pathological fractures, spinal cord compression, radiation or surgery to bone, and hypercalcemia of malignancy. Incidentally found radiographic SREs were identified through routine imaging studies, such as X-rays, CT scans, or MRIs, which were performed as part of the standard follow-up protocol for patients with bone metastases.

### 4.2 Statistical analysis

The primary endpoint of the study was to compare the time to first SRE in patients treated with ZA or DMAB. Time to first SRE was defined as the interval between the initiation of treatment to the occurrence of the first SRE, which encompassed pathological fractures, bone radiotherapy, spinal cord compression, and bone surgery. Secondary objectives included the assessment of progression-free survival (PFS) and overall survival (OS).

To mitigate confounding effects and imbalanced clinicopathological characteristics in patients treated with ZA or DMAB, we used propensity score matching (PSM) and the inverse probability of treatment weighting (IPTW) method. The PSM score was derived from logistic regression, with the BMA as the dependent variable and baseline variables as covariates. A 1:1 nearest neighbor matching with a caliper of 0.1 was implemented to establish matched cohorts. IPTW methods assigned weights to patients, forming pseudo-populations where treatment allocation was independent of covariates. The probabilities of receiving a specific BMA were calculated using a logistic regression model, with BMA treatment as the outcome and baseline clinical characteristics as covariates. Individual weights were computed based on the inverse probabilities of receiving the assigned treatment.

Baseline patient characteristics were compared using the chi-square (χ^2^) and Fisher's exact tests for categorical variables, and the *t*-test for continuous variables. The nonparametric Mann–Whitney *U* test was employed when the normality assumption was violated. The median follow-up time was estimated using the reverse Kaplan–Meier method.

To evaluate the influence of covariates on survival endpoints, we fitted unweighted (univariate) and weighted (multivariable) Cox proportional hazards regression models. Hazard ratios (HRs) and their 95 % confidence intervals (95 % CIs) were calculated to assess the proportional hazards assumption in the final Cox models. The dataset contained no missing values. A p-value of 0.05 or lower was deemed statistically significant. PSM and IPTW analyses were conducted using the RStudio Addins and Shiny Modules for Medical Research package, as well as the Jamovi software suite, version 1.6 [31,32].

## CRediT authorship contribution statement

**Roberta Scafetta:** Writing – original draft, Investigation, Conceptualization. **Marco Donato:** Writing – original draft, Investigation. **Carla Gullotta:** Investigation. **Alessandra Guarino:** Investigation. **Cristina Fiore:** Investigation. **Luisana Sisca:** Investigation. **Elena Speziale:** Investigation. **Raffaella Troiano:** Investigation. **Simone Foderaro:** Investigation. **Valentina Ricozzi:** Investigation. **Michele Iuliani:** Writing – original draft, Visualization. **Sonia Simonetti:** Writing – original draft, Visualization. **Silvia Cavaliere:** Visualization. **Alessio Cortellini:** Writing – review & editing. **Andrea Botticelli:** Writing – review & editing. **Simone Scagnoli:** Investigation. **Simona Pisegna:** Investigation. **Carmen Criscitiello:** Writing – review & editing. **Rebecca Pedersini:** Investigation. **Caterina Sposetti:** Investigation. **Elisa Tiberi:** Investigation. **Giuliana D'Auria:** Writing – review & editing. **Matteo Vergati:** Investigation. **Marco Mazzotta:** Investigation. **Roberta Caputo:** Investigation. **Annarita Verrazzo:** Investigation. **Maria Grazia Rossino:** Investigation. **Federica Domati:** Writing – review & editing. **Claudia Piombino:** Writing – review & editing. **Francesca Sofia Di Lisa:** Investigation. **Lorena Filomeno:** Investigation. **Teresa Arcuri:** Investigation. **Federica Puce:** Investigation. **Federica Riva:** Investigation. **Michela Palleschi:** Investigation. **Marianna Sirico:** Investigation. **Marta Piras:** Investigation. **Luigia Stefania Stucci:** Investigation. **Delia De Lisi:** Investigation. **Paolo Orsaria:** Investigation. **Edy Ippolito:** Investigation. **Sara Ramella:** Supervision. **Luca Visani:** Investigation. **Niccolò Bertini:** Investigation. **Ilaria Bonaparte:** Investigation. **Stefania Gori:** Writing – review & editing. **Luigi Rossi:** Writing – review & editing. **Icro Meattini:** Writing – review & editing. **Barbara Tagliaferri:** Writing – review & editing. **Orazio Caffo:** Supervision. **Ilaria Portarena:** Investigation. **Azzurra Irelli:** Investigation. **Elisabetta Cretella:** Writing – review & editing. **Camillo Porta:** Writing – review & editing, Supervision. **Giampaolo Bianchini:** Writing – review & editing, Supervision. **Agnese Fabbri:** Writing – review & editing, Supervision. **Ugo De Giorgi:** Supervision. **Patrizia Vici:** Supervision. **Angela Toss:** Supervision. **Ornella Garrone:** Writing – review & editing, Supervision. **Michelino De Laurentiis:** Writing – review & editing, Supervision. **Federica Villa:** Writing – review & editing, Supervision. **Rossana Berardi:** Writing – review & editing, Supervision. **Mauro Minelli:** Supervision. **Claudio Vernieri:** Writing – review & editing, Supervision. **Giuseppe Curigliano:** Writing – review & editing, Supervision. **Bruno Vincenzi:** Supervision. **Giuseppe Tonini:** Supervision. **Daniele Santini:** Writing – review & editing, Supervision. **Francesco Pantano:** Supervision, Methodology, Formal analysis, Conceptualization.

## Data availability

The data generated in this study are available within the article and its supplementary data files.

## Funding sources

This research did not receive any specific grant from funding agencies in the public, commercial, or not-for-profit sectors.

## Declaration of competing interest

The Authors declare no Competing Financial or Non-Financial Interests related to this study.

## References

[1] Cronin K.A. (2018). Annual report to the nation on the Status of cancer, part I: national cancer statistics. Cancer.

[2] Coleman R. (2007). Potential use of bisphosphonates in the prevention of metastases in early-stage breast cancer. Clin Breast Cancer.

[3] Coleman R.E. (2006). Clinical features of metastatic bone disease and risk of skeletal morbidity. Clin Cancer Res.

[4] D'Oronzo S., Coleman R., Brown J., Silvestris F. (2019). Metastatic bone disease: pathogenesis and therapeutic options: up-date on bone metastasis management. J Bone Oncol.

[5] Baron R., Ferrari S., Russell R.G. (2011). Denosumab and bisphosphonates: different mechanisms of action and effects. Bone.

[6] Clemons M., Gelmon K.A., Pritchard K.I., Paterson A.H. (2012). Bone-targeted agents and skeletal-related events in breast cancer patients with bone metastases: the state of the art. Curr Oncol.

[7] Wang L., Fang D., Xu J., Luo R. (2020). Various pathways of zoledronic acid against osteoclasts and bone cancer metastasis: a brief review. BMC Cancer.

[8] Santini D. (2003). Zoledronic acid induces significant and long-lasting modifications of circulating angiogenic factors in cancer patients. Clin Cancer Res.

[9] von Moos R. (2019). Management of bone health in solid tumours: from bisphosphonates to a monoclonal antibody. Cancer Treat Rev.

[10] Goetz M.P. (2017). Monarch 3: abemaciclib as initial therapy for advanced breast cancer. J Clin Oncol.

[11] Sledge G.W.J. (2017). Monarch 2: Abemaciclib in combination with fulvestrant in women with HR+/HER2- advanced breast cancer who had progressed while receiving endocrine therapy. J Clin Oncol.

[12] Sledge G.W. (2020). The effect of Abemaciclib plus fulvestrant on overall survival in hormone receptor-positive, ERBB2-negative breast cancer that progressed on endocrine therapy - MONARCH 2: a randomized clinical trial. JAMA Oncol.

[13] Hortobagyi G.N. (2022). Overall survival with Ribociclib plus letrozole in advanced breast cancer. N Engl J Med.

[14] Slamon D.J. (2020). Overall survival with Ribociclib plus fulvestrant in advanced breast cancer. N Engl J Med.

[15] Finn R.S. (2016). Palbociclib and letrozole in advanced breast cancer. N Engl J Med.

[16] Turner N.C. (2018). Overall survival with Palbociclib and fulvestrant in advanced breast cancer. N Engl J Med.

[17] Rugo H.S. (2019). Palbociclib plus letrozole as first-line therapy in estrogen receptor-positive/human epidermal growth factor receptor 2-negative advanced breast cancer with extended follow-up. Breast Cancer Res Treat.

[18] Stopeck A.T. (2010). Denosumab compared with zoledronic acid for the treatment of bone metastases in patients with advanced breast cancer: a randomized, double-blind study. J Clin Oncol.

[19] Lipton A. (2012). Superiority of denosumab to zoledronic acid for prevention of skeletal-related events: a combined analysis of 3 pivotal, randomised, phase 3 trials. Eur J Cancer.

[20] Ishikawa T. (2023). Differences between zoledronic acid and denosumab for breast cancer treatment. J Bone Miner Metabol.

[21] Fizazi K. (2011). Denosumab versus zoledronic acid for treatment of bone metastases in men with castration-resistant prostate cancer: a randomised, double-blind study. Lancet.

[22] Jiang L. (2021). Comparison of denosumab and zoledronic acid for the treatment of solid tumors and multiple myeloma with bone metastasis: a systematic review and meta-analysis based on randomized controlled trials. J Orthop Surg Res.

[23] Henry D.H. (2011). Randomized, double-blind study of denosumab versus zoledronic acid in the treatment of bone metastases in patients with advanced cancer (excluding breast and prostate cancer) or multiple myeloma. J Clin Oncol.

[24] Feng R. (2009). Targeting CDK4/CDK6 impairs osteoclast progenitor pool expansion and blocks osteolytic lesion development in multiple myeloma. ASH Annual Meeting Abstracts. Blood.

[25] Santo L., Siu K.T., Raje N. (2015). Targeting cyclin-dependent kinases and cell cycle progression in human cancers. Semin Oncol.

[26] Talluri S., Dick F.A. (2012). Regulation of transcription and chromatin structure by pRB: here, there and everywhere. Cell Cycle.

[27] Sherr C.J., Beach D., Shapiro G.I. (2016). Targeting CDK4 and CDK6: from discovery to therapy. Cancer Discov.

[28] Iuliani M. (2022). Biological effects of cyclin-dependent kinase inhibitors Ribociclib, Palbociclib and Abemaciclib on breast cancer bone microenvironment. Int J Mol Sci.

[29] Wang L. (2020). Various pathways of zoledronic acid against osteoclasts and bone cancer metastasis: a brief review. BMC Cancer.

[30] Perrone F. (2019). Adjuvant zoledronic acid and letrozole plus ovarian function suppression in premenopausal breast cancer: HOBOE phase 3 randomised trial. Eur J Cancer.

[31] Ho D., Imai K., King G., Stuart E.A. (2011). MatchIt: nonparametric preprocessing for parametric causal inference. J Stat Software.

[32] The jamovi project (2024). jamovi. https://www.jamovi.org.

