# Supplementary material for: Comparative analysis of Denosumab and Zoledronic acid in advanced breast cancer patients receiving CDK4/6 inhibitors
Source: Breast. 2025 May 15;82:104502. doi: 10.1016/j.breast.2025.104502 (PMC12152862; doi:10.1016/j.breast.2025.104502)
Supplement: Multimedia component 1 [file mmc1.pdf]

Supplementary Table: Univariate analysis

**Premenopausal State**	No	381 (73.7%)	-
	Yes	136 (26.3%)	0.90 (0.64-1.25, p=0.515)
**PS**	0	757 (87.6%)	-
	**1**	**107 (12.4%)**	**1.51 (1.12-2.03, p=0.007)**
**Histology**	Ductal	608 (70.4%)	-
	Lobular	194 (22.5%%)	1.05 (0.82-1.35, p=0.707)
	**Other**	**62 (7.2%)**	**1.60 (1.13-2.26, p=0.008)**
**Grading**	G1-G2	425 (65.2%)	-
	G3	227 (34.8%)	1.05 (0.81-1.37, p=0.699)
**Metastatic At Diagnosis**	No	305 (59.0%)	-
	Yes	212 (41.0%)	1.04 (0.78-1.39, p=0.777)
**Adjuvant Chemotherapy**	No	517 (60.8%)	-
	Yes	333 (39.2%)	0.81 (0.66-1.01, p=0.062)
**Adjuvant Endocrine**	No	320 (39.8%)	-
	Yes	485 (60.2%)	1.13 (0.91-1.41, p=0.257)
**Bone Only**	No	389 (45.0%)	-
	Yes	475 (55.0%)	0.93 (0.76-1.15, p=0.515)
**CDK4/6 Inhibitors**	Palbociclib	488 (56.5%)	-
	Ribociclib	253 (29.3%)	0.83 (0.66-1.06, p=0.130)
	Abemaciclib	123 (14.2%)	0.83 (0.58-1.18, p=0.290)
**Line of Treatment**	First	678 (80.2%)	-
	Second	167 (19.8%)	0.85 (0.65-1.13, p=0.261)
**Setting**	Endocrine Resistant	366 (42.4%)	-
	Endocrine Sensitive	498 (57.6%)	0.94 (0.76-1.15, p=0.538)
**Endocrine Therapy**	Aromatase Inhibitor	535 (61.9%)	-
	Fulvestrant	329 (38.1%)	1.05 (0.85-1.31, p=0.632)
**Age**	Mean (± SD)	60.6 (12.6%)	1.01 (1.00-1.02, p=0.211)
**Ki67**	Mean (± SD)	23.1 (14.7%)	1.00 (0.99-1.01, p=0.857)
**Estrogen Receptor**	Mean (± SD)	86.6 (15.2%)	0.99 (0.98-1.00, p=0.143)
**Progesterone Receptor**	Mean (± SD)	48.7 (35.9%)	1.00 (1.00-1.00, p=0.899)
